# Toxin-antitoxin loci *vapBC-1* and *vapXD* contribute to survival and virulence in nontypeable *Haemophilus influenzae*

**DOI:** 10.1186/1471-2180-12-263

**Published:** 2012-11-19

**Authors:** Dabin Ren, Anna N Walker, Dayle A Daines

**Affiliations:** 1 Division of Basic Medical Sciences, Mercer University School of Medicine, Macon, GA, USA; 2 Department of Pathology, Mercer University School of Medicine, Macon, GA, USA; 3 Department of Biological Sciences, Old Dominion University, Norfolk, VA, USA; 4 Current address: Rochester General Hospital Research Institute, Rochester, NY, USA

**Keywords:** NTHi, Otitis media, Protein-protein interactions, Stress, Ribonuclease

## Abstract

**Background:**

Nontypeable *Haemophilus influenzae* (NTHi) is a significant human pathogen responsible for respiratory tract infections and the most common cause of recurrent otitis media. Type II toxin-antitoxin (TA) systems are genetic elements that code for a stable protein toxin and a labile antitoxin that are thought to be involved in metabolic regulation of bacteria by enabling a switch to a dormant state under stress conditions. The contribution to infection persistence of the NTHi TA loci *vapBC-1* and *vapXD* was examined in this study.

**Results:**

Deletions in *vapBC-1, vapXD* and *vapBC-1 vapXD* significantly decreased the survival of NTHi co-cultured with primary human respiratory tissue at the air-liquid interface and in the chinchilla model of otitis media. The TA deletions did not affect the growth dynamics of the mutants in rich media, their ultra-structural morphology, or display appreciable synergy during NTHi infections. The toxin and antitoxin proteins of both pairs heterodimerized *in vivo*. Consistent with our previous findings regarding the VapC-1 toxin, the NTHi VapD toxin also displayed ribonuclease activity.

**Conclusions:**

We conclude that the *vapBC-1* and *vapXD* TA loci enhance NTHi survival and virulence during infection *in vitro* and *in vivo* using a mechanism of mRNA cleavage, and that these conserved TA pairs represent new targets for the prophylaxis and therapy of otitis media and other NTHi-caused mucosal diseases.

## Background

Nontypeable *Haemophilus influenzae* (NTHi) is a Gram-negative organism that is both a common commensal of the upper respiratory tract as well as a significant cause of respiratory tract infections in humans. NTHi is the second most common cause of acute otitis media after *Streptococcus pneumoniae* and, in many studies, is the most common cause of recurrent otitis media based on cultures of middle ear fluids obtained by tympanocentesis [1]. Recurrent otitis media is associated with pain, the need for insertion of tympanostomy tubes under general anesthesia, conductive hearing impairment, and delayed speech and language development [2]. Currently, otitis media is commonly treated with antibiotics, among which amoxicillin is the consensus recommendation for the initial therapy [3,4]. But approximately 20–35% of NTHi strains, depending on geographic location, produce β-lactamase and these strains are resistant to amoxicillin [4]. Moreover, there is currently no licensed vaccine available to prevent NTHi infections. Thus, illuminating the molecular mechanisms of NTHi infections could lead to the development of novel strategies to improve prophylaxis and treatment of otitis media.

Adhesin molecules on the surface of NTHi are shown to bind to respiratory tract target cells and activate these cells to induce inflammation [5,6]. NTHi also penetrates into human respiratory tract cells (epithelial cells and macrophages) and the interstitium to cause nasopharyngeal colonization and respiratory infection [7-10]. Biofilms of NTHi found in middle ears are postulated to be responsible for the resistance to clearance by host immune responses and antibiotic treatments, therefore resulting in recurrent otitis media [5,6,11,12]. However, there is controversy whether the reported biofilm is an outcome of infectious interactions between the host and NTHi or a programmed phenotype of NTHi virulence [13]. Although these observations have advanced our understanding, much of the pathogenesis of NTHi-induced otitis media, especially recurrent otitis media, is largely unknown.

Toxin-antitoxin (TA) systems are small genetic modules comprised of two components, a stable toxin and its labile antitoxin. TA systems in prokaryotic genomes are classified into 3 types, based on the antitoxin nature and mode of action. While toxins are always proteins, antitoxins are either RNAs (types I and III) or proteins (type II) [14]. Several common families of type II modules have been identified on the chromosomes of bacteria and archaea: *relBE*, *higBA*, *mazEF*, *ccdAB*, *vapBC*, *parDE*, *phd–doc*, ζε, *hipBA*, and *yoeB–yefM*[15]. Type II TA systems are thought to be part of the mobilome and to move from one genome to another through horizontal gene transfer [16,17]. They are hypothesized to function in stress management either by promoting altruistic sacrifice of a large fraction of the population (the programmed cell death hypothesis, PCD) or by inducing a dormant stage (bacteriostasis) that allows cells to cope with stress [18-20].

Type II TA systems are typically two-gene operons with the antitoxin encoded upstream of the toxin gene. The proteic antitoxins bind their cognate toxins and inhibit toxin activity. Antitoxins or toxin-antitoxin complexes autorepress TA module transcription. Under stressful environmental conditions such as nutrient limitation, antibiotic therapy, or oxidative stress, TA modules are activated. The labile antitoxin is degraded by either the Lon or Clp proteases and the more stable toxin is freed to facilitate growth arrest. Many toxins are mRNA-specific RNases that rapidly inhibit protein synthesis, inducing a bacteriostatic state. Upon improved conditions (or removal of stress), antitoxin synthesis resumes to counteract toxin activity, and tmRNA activity rescues ribosomes arrested on toxin-cleaved messages [21,22]. Virulence-associated protein (*vap*) genes, first identified in pathogenic strains of the Gram-negative, strict anaerobe *Dichelobacter nodosus*, are found as transmissible genetic elements for the transfer of virulence determinants [23]. The *vap* genes are recognized as a part of pathogenicity islands (PAI), a group of laterally-transferred genes in the bacterial genome, which help the organism explore and adapt to new ecological niches [24,25].

Four *vap* operons, *toxAvapA*, *vapBC-1*, *vapBC-2*, and *vapXD* have been identified in the genomes of numerous NTHi strains, including Rd KW20 [26], R2866 [27], and 86-028NP [28]. All *vap* operons display the characteristic features of type II TA modules, and *vapBC-1* and *vapXD* have been shown to act as TA loci in NTHi [29,30]. During recurrent and chronic otitis media, NTHi are exposed to hostile conditions such as antibiotic treatment, host immune responses, and nutrient deprivation. It is thought that a subpopulation of NTHi can resume the infection after cessation of these stressors, resulting in a persistent infection. Although TA modules function to allow bacterial adaptation to environmental stresses, the pathogenic roles of the NTHi *vapBC-1* and *vapXD* operons have not been elucidated in otitis media. It has been shown that the protein products of canonical type II TA loci interact to form protein complexes that autoregulate their cognate promoters [22]. Accordingly, we examined the heterodimerization characteristics of the VapB-1 antitoxin with the VapC-1 toxin, as well as interactions of the antitoxin VapX with the toxin VapD. We then constructed *vapBC-1*, *vapXD*, and *vapBC-1 vapXD* double deletion mutants in strain 86-028NP. The survival properties of these mutants were compared to the wild type parent strain during long-term infections of a primary human respiratory epithelial tissue at the air-liquid interface (ALI), the EpiAirway™ tissue. The NTHi TA mutants were then analyzed for persistence *in vivo* using the chinchilla model of otitis media over the course of a 4-day infection. Finally, we tested the VapD toxin for ribonuclease activity *in vitro*. The current work is aimed at uncovering the contributions of *vapBC-1* and *vapXD* to NTHi-caused otitis media, which could lead to new vaccine or pharmaceutical targets for the prophylaxis and therapy of this disease.

## Results

### Interactions of the Vap proteins *in vivo*

**Figure 1:**
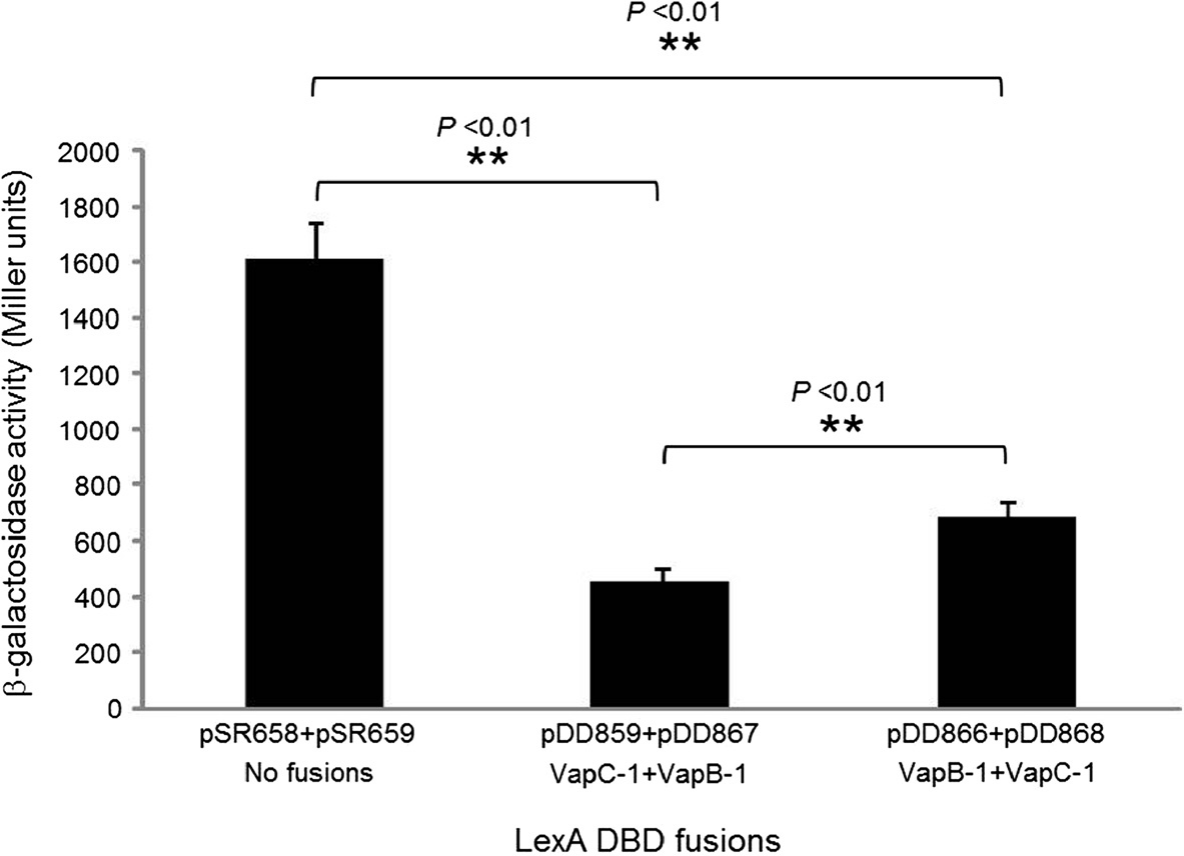
**VapB-1 and VapC-1 heterodimerize *in vivo*.** 86-028NP *vapB-1* or *vapC-1* was fused to the LexA DNA binding domain (DBD) in the vectors pSR658 or pSR659, resulting in pDD866 or pDD868, respectively. Reciprocally, *vapC-1* or *vapB-1* was also fused to the LexA DBD in the vectors pSR658 or pSR659, resulting in pDD859 or pDD867, respectively. Each pair was co-transformed into the reporter strain SU202 and the amount of heterodimerization was quantitated by β-galactosidase activity assays (n = 3 in triplicate). Data are expressed as mean ± SD.

To detect the ability of VapB-1 and VapC-1 to form heterodimers *in vivo*, β-galactosidase activity assays were carried out using an *E. coli*-based LexA protein-protein interaction reporter system as previously described [31]. In this system, with no protein fused to the LexA DNA binding domain (DBD) of either plasmid pSR658 or pSR659 in strain SU202, the repressor cannot form a dimer, and the expression of the *lacZ* reporter gene is constitutive. However, a reconstituted repressor formed by heterodimerization of fused proteins can bind to the engineered operator region, decreasing transcription of the reporter gene, but a homodimer, if formed, cannot bind to the operator. Since the LexA DBD plasmids have different copy numbers (pSR658 has a higher copy number than pSR659), we constructed reciprocal fusions and analyzed each set as an internal control for heterodimerization. When we fused VapC-1 to the LexA DBD in pSR658 (pDD859) and VapB-1 to pSR659 (pDD867), the reporter gene expression was decreased to 458 ± 47 Miller units, whereas the unfused LexA DBD in the vectors pSR658 and pSR659 allowed constitutive transcription of the reporter gene at 1,611 ± 138 Miller units (Figure 1). This indicated a strong protein:protein interaction. When the fusions were reversed, with VapB-1 in pSR658 (pDD866) and VapC-1 in pSR659 (pDD868), the pair heterodimerized and repressed *lacZ* expression to 682 ± 61 Miller units. Interestingly, there was a significant difference between the reciprocal fusions, with the pDD859/pDD867 pair being the most efficient at repressing the reporter gene.

**Figure 2:**
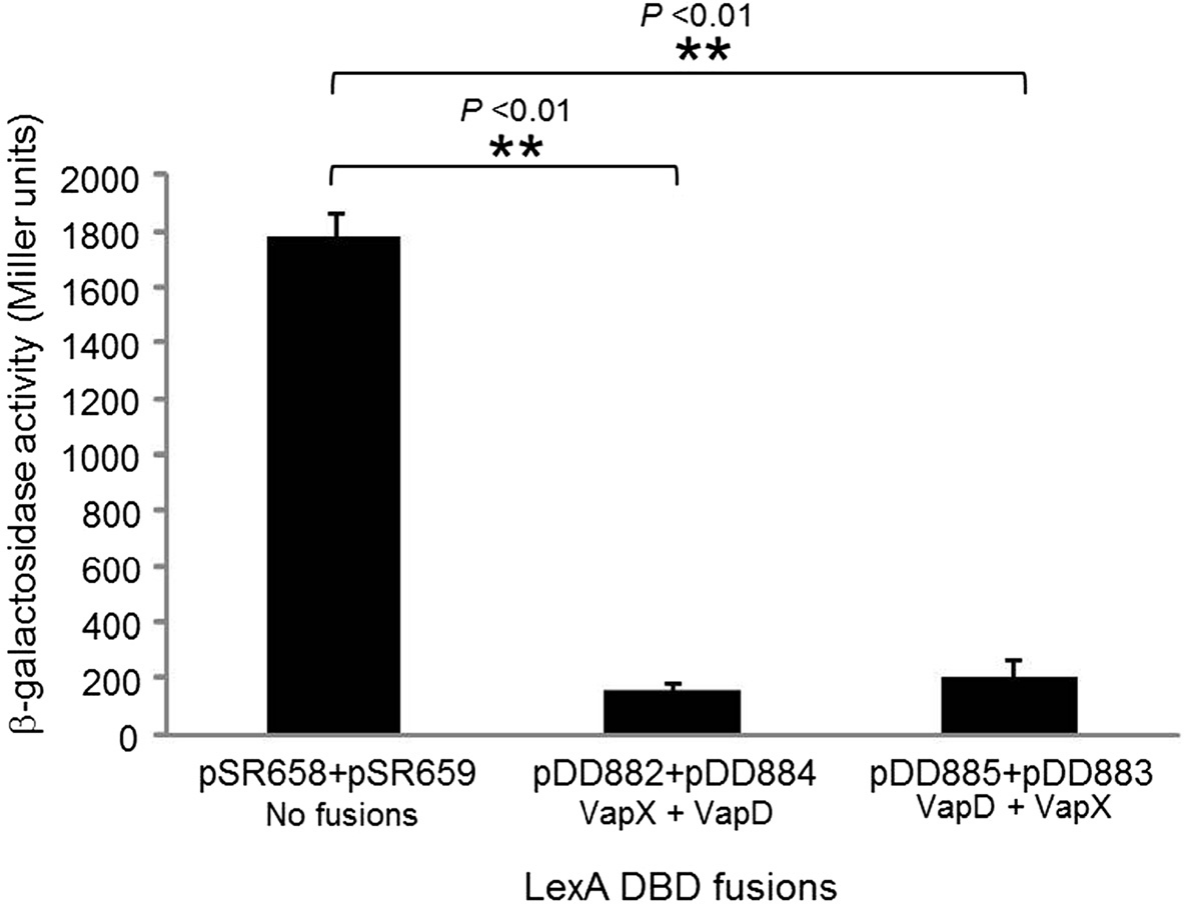
**VapX and VapD heterodimerize *in vivo.*** 86-028NP *vapX* or *vapD* was fused to the LexA DNA binding domain (DBD) in the vectors pSR658 or pSR659, resulting in pDD882 or pDD884, respectively. Reciprocally, *vapD* or *vapX* was also fused to the LexA DBD in the vectors pSR658 or pSR659, resulting in pDD885 or pDD883, respectively. Each pair was co-transformed into the reporter strain SU202 and the amount of heterodimerization was quantitated by β-galactosidase activity assays (n = 3 in triplicate). Data are expressed as mean ± SD.

To investigate the hetero-interactions between VapX and VapD, the same reporter system was used as above. With VapX in pSR658 (pDD882) and VapD in pSR659 (pDD884), the reporter gene expression was decreased to 162 ± 27 Miller units, compared to the expression in the presence of the control vectors of 1,783 ± 85 Miller units (Figure 2). Likewise, the reciprocal fusion, with VapD in pSR658 (pDD885) and VapX in pSR659 (pDD883), resulted in 210 ± 61 Miller units, both significantly lower than the vector control. In contrast to VapB-1 and VapC-1, no significant difference was observed between the reciprocal fusions for VapX and VapD heterodimerization.

### Growth dynamics of cultivated NTHi mutants

**Figure 3:**
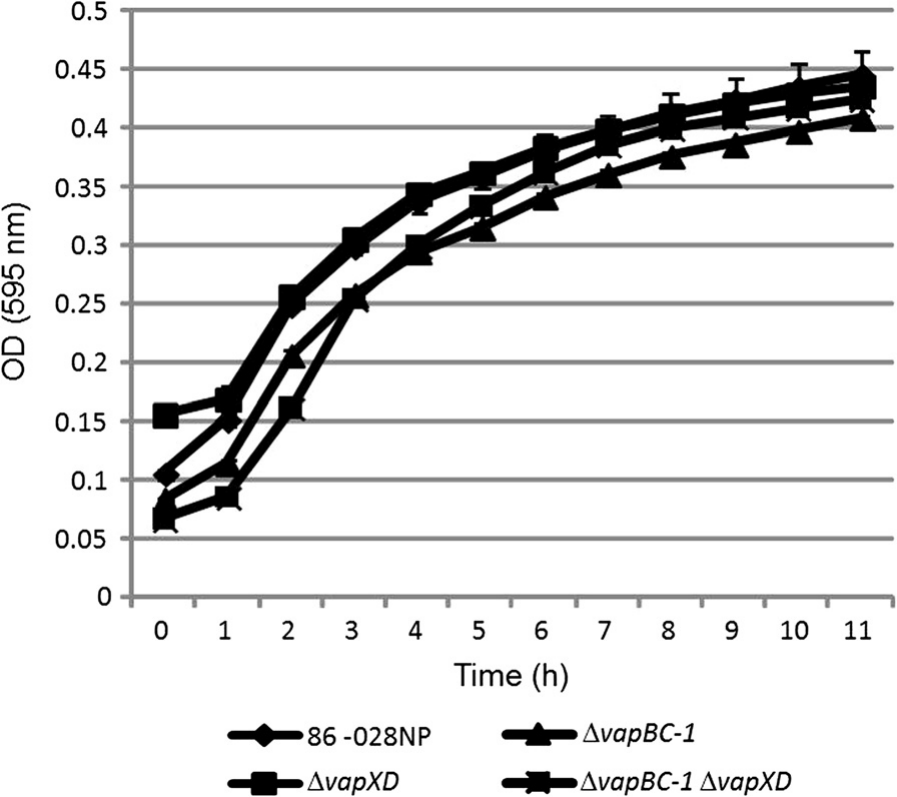
**Growth dynamics of the parent strain and *vap *mutants.** Strain 86-028NP and the Δ*vapBC-1*, Δ*vapXD*, and Δ*vapBC-1* Δ*vapXD* mutants were grown in a 96 well plate at 35°C with shaking (n = 2 in triplicate) to analyze any differences in replication. Data are expressed as mean ± SD. No significant difference between the growth dynamics of the various strains was observed.

The growth behavior of the 86-028NP parent strain and the Δ*vapBC-1*, Δ*vapXD*, and Δ*vapBC-1* Δ*vapXD* mutants was evaluated by culturing in sBHI for 11 h (Figure 3). The bacterial numbers of all the strains increased most rapidly during the first 5 hours of culture, followed by entry into stationary phase. No significant difference in growth dynamics was observed between the strains, demonstrating that any differences between the survival of the wild type parent strain and the mutants in primary human respiratory tissues or the chinchilla middle ear model was not attributable to a defect in replication under normal culture conditions.

### Ultrastructure of NTHi mutants co-cultured with EpiAirway tissues

**Figure 4:**
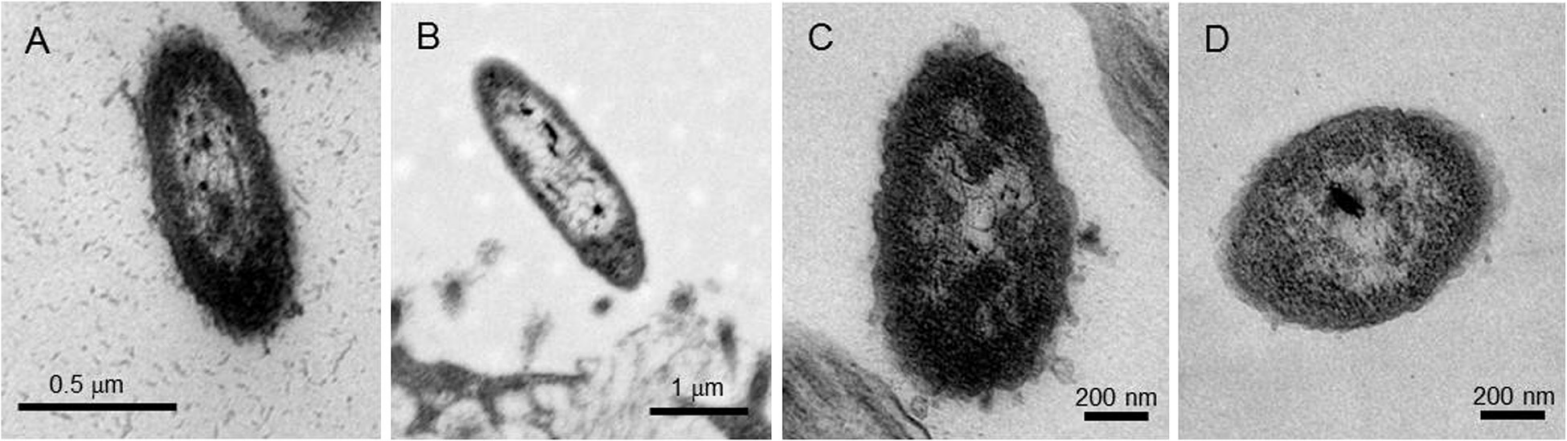
**Ultra-structure of NTHi mutants co-cultured with EpiAirway tissues.** EpiAirway tissues were infected with the wild type (**A**), Δ*vapBC-1* (**B**), Δ*vapXD* (**C**), or Δ*vapBC-1* Δ*vapXD* (**D**) strains at ~10^7^ colony forming units (CFU) per insert. On day 5 after infection, the tissues were fixed and sectioned for transmission electron microscopy. No significant difference in morphology was observed for any of the mutants.

To assess the effects of the TA loci on the morphologic aspects of NTHi invasion behavior, a primary human respiratory epithelial tissue model at the ALI, the EpiAirway, (MatTek, Ashland, MA USA) was used in long-term co-culture with the various strains. Ultrastructure of the NTHi strains was observed by TEM on day 5 post-infection (Figure 4). The 86-028NP parent strain (Figure 4A), Δ*vapBC-1* (Figure 4B), Δ*vapXD* (Figure 4C), and Δ*vapBC-1* Δ*vapXD* mutants (Figure 4D) all were found residing both apically and within the tissues. Although NTHi are pleomorphic by nature, the mutant organisms associated with the tissues were intact and no significant structural damage was observed in any of the mutant strains.

### Attenuation of the *vap* mutants in the EpiAirway tissue model

**Figure 5:**
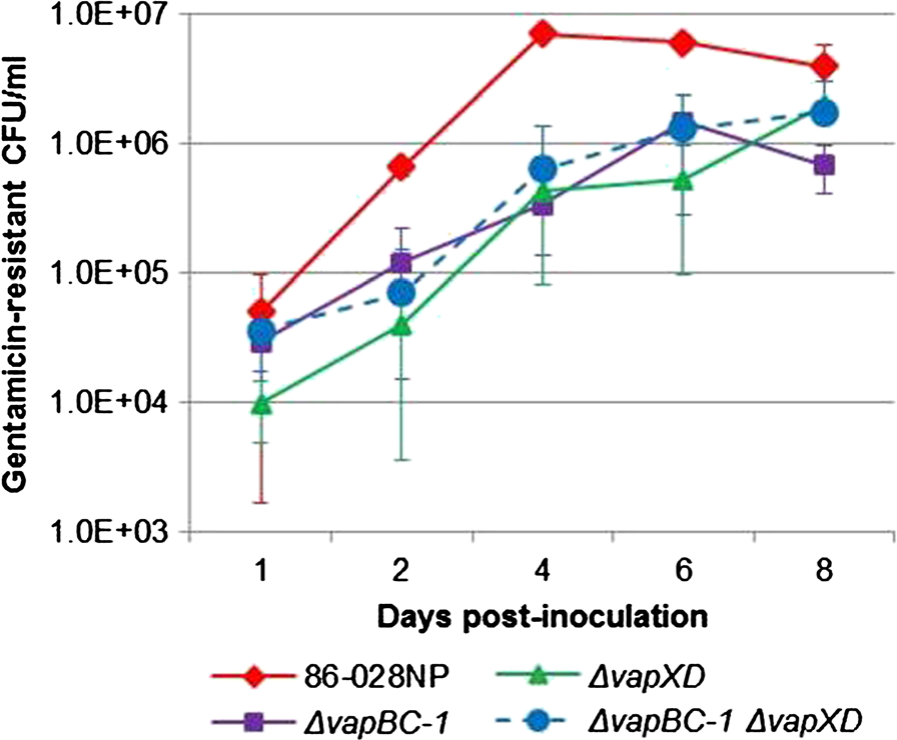
**NTHi mutants are attenuated during long-term co-culture in the EpiAirway tissue model.** EpiAirway tissues (n = 6) were infected with 86-028NP wild type, Δ*vapBC-1*, Δ*vapXD*, or Δ*vapBC-1* Δ*vapXD* mutants at ~10^7^ colony forming units (CFU) per insert. On days 1, 2, 4, 6, and 8 after infection, gentamicin-resistant bacteria were harvested for CFU counts. Data are expressed as mean ± SD.

The EpiAirway model consists of primary, well-differentiated tissues with tight junctions, ciliated and nonciliated cells, mucin-producing goblet cells, and is an excellent *in vitro* representation of the human upper airway [32]. Indeed, the transcriptional profile of respiratory epithelial cells cultured at the ALI has been shown to closely resemble that of the *in vivo* airway epithelium [33]. To determine the contribution of the *vapBC-1* and *vapXD* TA loci to NTHi survival capability within primary human tissues, the 86-028NP wild type, the Δ*vapBC-1*, Δ*vapXD*, and Δ*vapBC-1* Δ*vapXD* mutants were co-cultured with the EpiAirway tissues and the number of internalized (gentamicin-resistant) bacteria for each strain was enumerated over 8 days of co-culture (Figure 5). Although each strain was inoculated at ~10^7^ CFU, the number of internalized wild type bacteria (86-028NP) was greater for all time points than those of the Δ*vapBC-1*, Δ*vapXD*, or Δ*vapBC-1* Δ*vapXD* mutant strains, which showed significantly lower survival levels over the 8 days of co-culture (n = 6, *P* < 0.05).

### The *vap* mutants are attenuated in the chinchilla otitis media model

**Figure 6:**
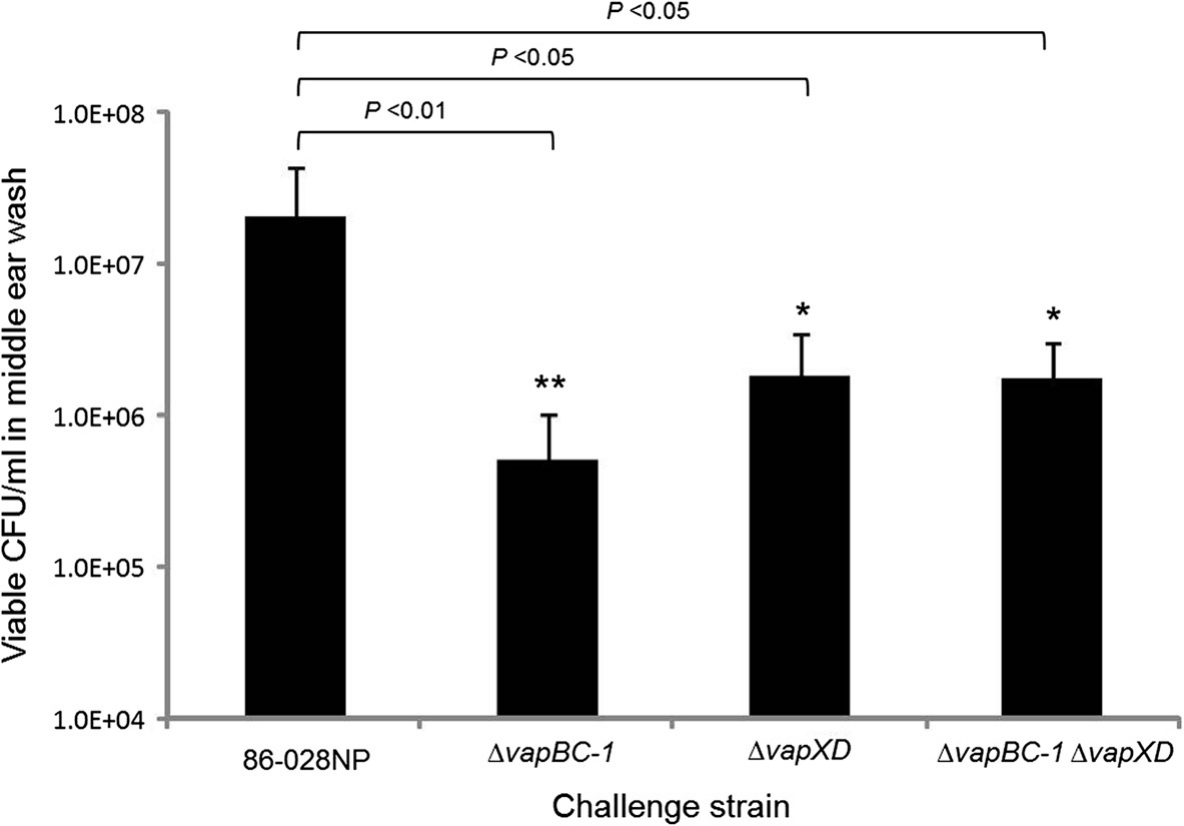
**NTHi mutants are attenuated in the chinchilla otitis media model.** Chinchillas (4–5 animals representing 8–10 middle ears per challenge strain) were transbullarly injected with 100 μl (~ 1000 CFU) of the 86-028NP wild type, Δ*vapBC-1*, Δ*vapXD*, or the Δ*vapBC-1* Δ*vapXD* mutant strain, respectively. On day 4 post-challenge, the middle ears were washed and bacterial CFU counts were obtained. Data are expressed as mean ± SD.

The chinchilla model of otitis media was employed to determine the survival of the NTHi mutants over the course of a 4-day infection (Figure 6). After 4 days, an average of 2.1 × 10^7^ CFU/ml of the 86-028NP parent strain was recovered from chinchilla middle ears. In contrast, the Δ*vapBC-1*, Δ*vapXD*, and Δ*vapBC-1* Δ*vapXD* mutants recovered from the infected middle ears were an average of 5.1 × 10^5^, 1.8 × 10^6^, and 1.8 × 10^6^ viable CFU/ml, respectively, all significantly lower than the wild type strain (*n* = 8–9 ears, *P* < 0.05). The Δ*vapBC-1* mutant exhibited the lowest recovery numbers from the infected middle ears among all the tested strains (*n* = 8 ears, *P* < 0.05). No significant difference between the recovered CFU numbers was observed for the Δ*vapXD* single mutant and the Δ*vapBC-1* Δ*vapXD* double mutant strain (Figure 6).

### The *vap* mutants elicited lower levels of inflammation

**Figure 7:**
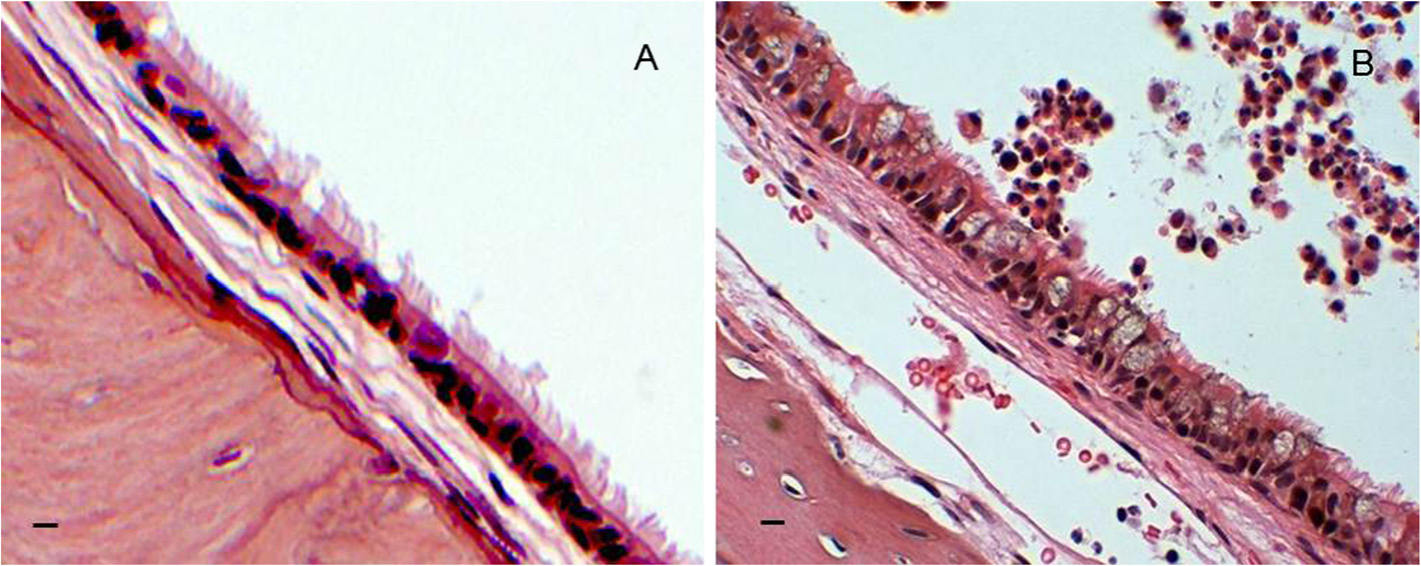
**Chinchilla middle ear sections from control and infected animals.** Representative H&E stained sections from **A**) PBS control (inflammatory score = 0) and **B**) 86-028NP-infected (inflammatory score = 4+) animals. Scale bars are 10 μm.

It has been shown that even nonviable NTHi (e.g. a whole bacterial cell lysate) can induce an immune response in middle ear cells *in vitro* and *in vivo*[34]. To determine if the numbers of viable organisms recovered from the chinchilla middle ears correlated with the inflammatory response, we sagittally step-sectioned middle ears of animals infected with either the wild type parent or each of the various mutant strains. Subsequent hematoxylin-eosin (H&E) stains of each ear were randomized and blinded, then scored by one of us (A.N.W., a Board-certified pathologist) for the extent of inflammation using a scale from 0 (no inflammation, PBS control) to 4+ (greatest inflammatory response observed). Examples of PBS control (A, inflammatory score = 0) and 86-028NP infected (B, inflammatory score = 4+) H&E-stained chinchilla middle ears are shown in Figure 7. Consistent with the numbers of viable bacteria recovered, the middle ear sections from animals infected with the mutant strains exhibited less inflammation on average than the wild type parent strain (Table 1). This suggests that the *vap* mutants were killed and cleared earlier in the infection process, supporting both the role of these TA operons in the pathogenesis of otitis media and the importance of these modules as new therapeutic targets.

**Table 1:** Inflammatory response scores of chinchilla middle ear sections

**Strain**	**Inflammatory score**^**a**^			
	**1+**	**2+**	**3+**	**4+**
86-028NP	1	2	4	1
Δ*vapBC-1*	1	6	1	0
Δ*vapXD*	2	4	2	0
Δ*vapBC-1* Δ*vapXD*	4	4	0	0

^a^8 middle ears were scored for each challenge strain.

### VapD displays ribonuclease activity

**Figure 8:**
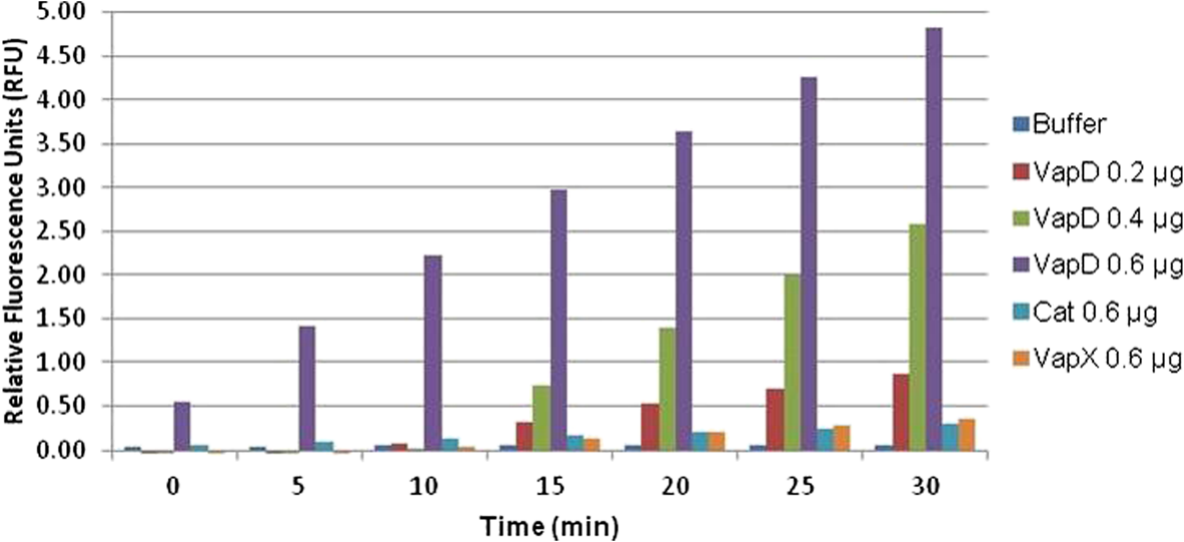
**RNase activity assays with purified VapD, Cat, and VapX.** Ribonuclease activity over time of the protein elution buffer control (blue), 0.2 μg (red), 0.4 μg (green), and 0.6 μg (purple) of purified VapD, 0.6 μg of chloramphenicol acetyltransferase (Cat, turquoise), or 0.6 μg of VapX (orange) assayed with the RNaseAlert test kit (IDT, Coralville, IA). Indicated amounts of proteins were added to 25 pmol fluorophore-conjugated RNaseAlert substrate. The substrate has a quencher on one end and a fluorophore (FAM) on the other. Cleavage of the single-stranded RNA removed the quencher and the resulting fluorescence was read on a MiniOpticon real-time detection system. The Cat protein and the VapX antitoxin were overexpressed and purified in an identical fashion to VapD and serve as negative protein controls.

We have previously shown that VapC-1 is a ribonuclease [30]. Since the Δ*vapXD* mutant was also attenuated for survival *in vitro* and *in vivo*, we assayed the purified VapD toxin for RNase activity, and found that it was a potent ribonuclease (Figure 8). These data are consistent with a recent publication that demonstrated the ribonuclease activity of a VapD homologue from *Helicobacter pylori*[35]. Figure 8 shows a RNase activity assay conducted over time using the RNaseAlert (Integrated DNA Technologies, Coralville, IA) substrate with increasing amounts of VapD protein. The single-stranded RNA substrate has a quencher on one end and a fluorophore (FAM) on the other, and fluoresces brightly when cleaved. We included protein elution buffer, purified Cat (chloramphenicol acetyltransferase), and antitoxin VapX proteins as negative controls, which were overexpressed and purified in the identical fashion as VapD. The VapD protein displayed concentration-dependent RNase activity over time in this assay.

## Discussion

As classic type II TA partners, VapB-1 and VapC-1 were previously found to functionally interact in regulating the ribonuclease activity of NTHi VapC-1 *in vitro*[30]. Likewise, in another study, the presence of VapX was required to relieve the cell growth arrest caused by VapD [29]. Here we demonstrate with a LexA detection system that both protein pairs also physically interact *in vivo*. Based on the TA model hypothesis, these observations suggest that under favorable conditions, the antitoxins VapB-1 and VapX bind to and inhibit the toxins VapC-1 and VapD, respectively. During infections of NTHi-caused otitis media, various stress stimuli such as nutrient deprivation, antibiotics, and reactive oxygen species encountered by the organisms might result in the release of the VapC-1 and VapD toxins from their degraded or inactivated cognate antitoxins VapB-1 and VapX, respectively*.* The mobilization of these toxins could then trigger or facilitate downstream events such as mRNA decay of metabolism-related transcripts, driving the bacterial population into a stasis state and leading to a persistent infection of NTHi in the middle ear of the host.

Deletions of either or both of the *vapBC-1* and *vapXD* TA loci did not change the cellular morphology of the organism during co-culture as revealed by TEM examination, and the ability of the mutants to replicate normally in rich media was not affected. This indicates that the observed attenuation of persistence was not associated with detrimental changes in the morphologic structure or replication dynamics of the pathogen, but rather was attributable to the lack of the apparently protective effect of the *vap* pairs.

A common feature of type II TA systems is a toxic enzyme activity that switches bacterial cells over to metabolic stasis under stressful conditions such as starvation [36,37] as well as heat, osmotic and free radical-induced stress [38]. Indeed, VapC toxin homologues from *M. tuberculosis* inhibited growth when expressed without their cognate VapB antitoxins in *M. smegmatis*[39]. An obvious conclusion to be drawn from this conserved attribute is that, without the toxin present to facilitate a state of bacteriostasis, the organism could continue to replicate under conditions that would normally allow toxin activation followed by growth arrest. Our data suggest that the loss of the ability to modulate replication is detrimental to NTHi in our infection models. For example, the Δ*vapBC-1* mutant co-cultured with EpiAirway tissues displayed a marked attenuation of survival, suggesting that the *vapBC-1* locus contributed significantly to persistence under these conditions. Deletion of the *vapXD* locus or both *vapBC-1 vapXD* loci reduced NTHi persistence to similar levels when co-cultured with the EpiAirway tissues, indicating that the *vapXD* locus was also involved in maintaining the NTHi survival during extended infections. Interestingly, during the early (Day 1) and late (Day 8) time points, the differences between the wild type and mutant strains were less marked than during Days 2, 4, and 6. The reasons for this phenotype are unclear, but it may be due to unregulated replication of the *vap* mutants within the EpiAirway tissues, which could result in nutrient deprivation-induced death after the first 24 hours of infection. We have recently shown by TEM and immunoelectron microscopy that NTHi are often located between the basal cells in these tissues [40]. While the apical surfaces of infected tissues were undamaged, the basal cells displayed wider intercellular junctions and pockets of necrotic debris. This is consistent with the hypothesis that the late (Day 8) increases in mutant survival could be due to necrosis of a subset of basal respiratory epithelial cells, providing more nutrients to the *vap* mutants and allowing their numbers to approach that of the wild type strain.

Our *in vivo* results further confirmed the EpiAirway findings by showing that the survival of all three mutants was significantly decreased when compared to the wild type strain after a 4-day infection in the chinchilla model of otitis media. The double deletion of *vapBC-1* and *vapXD* did not increase the average attenuation of persistence in comparison to the single deletion of *vapXD* in either model. This lack of synergy suggests that neither locus serves as an agonist or antagonist for the other, but rather that each may act independently to modulate replication. Moreover, consistent with the numbers of viable bacteria recovered, the inflammatory scores of the middle ear sections were lower for the mutants than for the wild type strain, although the animals were able to mount an effective inflammatory response after infection.

Similar to our VapC-1 data [30], we show that NTHi VapD displays ribonuclease activity *in vitro*. This finding suggests that the toxins of both *vap* operons may play key roles in stress-induced post-transcriptional regulation of gene expression via the mechanism of mRNA cleavage. Taken together, our *in vitro* and *in vivo* data demonstrate that both the *vapBC-1* and *vapXD* TA loci function to maintain NTHi survival and virulence. This is the first report, to our knowledge, of the *vapBC-1* and *vapXD* loci playing a role in the pathogenesis of NTHi infections *in vivo*. Other conserved TA pairs have been suggested as novel antimicrobial targets [41], and our data support the notion that TA deletion results in detrimental effects on NTHi infection progression. Further studies are proposed to determine the most effective way of interfering with toxin activity *in vivo*.

## Conclusions

We conclude that the *vapBC-1* and *vapXD* TA loci enhance NTHi survival and virulence during infection *in vitro* and *in vivo* using a mechanism of mRNA cleavage, and that these conserved TA pairs represent new targets for the prophylaxis and therapy of otitis media and other NTHi-caused mucosal diseases.

## Methods

### Bacterial strains and culture conditions

The bacterial strains and plasmids used in these studies are listed in Table 2. *Escherichia coli* strains were grown in LB broth or agar with or without 30 μg/ml kanamycin, 100 μg/ml ampicillin, 20 μg/ml chloramphenicol, or 12 μg/ml tetracycline as required. For the protein-protein interaction studies, strain SU202 carrying both pSR658 and pSR659 (unfused control plasmids) or any of the tested protein fusions was grown overnight in LB broth containing 1 mM IPTG and then diluted and grown to log phase in LB containing 1 mM IPTG prior to β-galactosidase activity assays. NTHi strains were grown in brain heart infusion broth or agar supplemented with 10 μg/ml heme-histidine and 10 μg/ml β-NAD (sBHI). *E. coli* strain BL21 (DE3) was used to overexpress epitope-tagged Cat, VapD and VapX for protein purification prior to use in ribonuclease (RNase) activity assays. To construct the NTHi mutants, transformants were selected on chocolate agar plates with 30 μg/ml kanamycin or 2 μg/ml chloramphenicol as required. NTHi were routinely cultured at 37°C with 5% CO_2_.

**Table 2:** Bacteria and plasmids used in this study

**Strain**	**Description**	**Source**
86-028NP	NTHi nasopharyngeal isolate from a pediatric otitis media patient	R. S. Munson, Jr.
BL21(DE3)	*fhuA2 [lon] ompT gal (λ DE3) [dcm] ∆hsdS λ DE3 = λ sBamHIo ∆EcoRI-B int::(lacI::PlacUV5::T7 gene1) i21 ∆nin5*	Invitrogen
DH5α	λ―Φ80*ΔlacZΔM15 Δ(lacZYA-argF)U169 recA1 endA1 hsdR17(rK―mK―)supE44 thi-1 gyrA relA1*	Laboratory collection
SU202	*lexA71*::Tn*5*(Def)*sulA211*Δ(*IacIPOZYA*)*169*/F’*lacI*^q^*lacZM15*::Tn*9*	M. Granger-Schnarr
**Plasmid**	**Description**	**Source**
pGEM5	Cloning vector	Promega
pUC4K	Source of the kanamycin cassette	Lab collection
pBluescript II	Cloning vector	Lab collection
pUCΔE*cat*	Source of the chloramphenicol cassette	Lab collection
pET24b	Expression vector	Novagen
pSR658	LexA DBD fusion vector	[31]
pSR659	LexA DBD fusion vector	[31]
pDD689	pET24b with the *cat* gene	[30]
pDD731	pGEM5 with the *vapBC-1* gene deletion	This work
pDD788	pBluescript II SK(+) with the *vapXD* deletion	This work
pDD791	pET24b with *vapXD*	This work
pDD859	pSR658 with *vapC-1* fused to the LexA DBD	This work
pDD866	pSR658 with *vapB-1* fused to the LexA DBD	This work
pDD867	pSR659 with *vapB-1* fused to the LexA DBD	This work
pDD868	pSR659 with *vapC-1* fused to the LexA DBD	This work
pDD882	pSR658 with *vapX* fused to the LexA DBD	This work
pDD883	pSR659 with *vapX* fused to the LexA DBD	This work
pDD884	pSR659 with *vapD* fused to the LexA DBD	This work
pDD885	pSR658 with *vapD* fused to the LexA DBD	This work
pDD902	pET24b with *vapX*	This work

### Construction of NTHi mutants

NTHi 86-028NP Δ*vapBC-1*, Δ*vapXD*, and Δ*vapBC-1* Δ*vapXD* mutants were constructed by homologous recombination using MIV transformation as previously described [42]. For the Δ*vapBC-1* mutant construction, the *vapBC-1* gene region (2558 bp) was amplified from 86-028NP genomic DNA by high-fidelity PCR with primers BCXbaFor (5^′^-GCTTTCTAGACAGGCTAAATATACCG-3^′^) and BCXbaRev (5^′^-GGTCTCTAGAGGCATTGTGCGCCAC-3^′^) with engineered *XbaI* sites (underlined). The PCR product was cut with the restriction endonuclease *XbaI* and cloned into pGEM5 cut with *SpeI*, resulting in pDD747. This plasmid was then cut with *BamHI* and *BglII* and gel-purified, creating a 564 bp deletion in the 636 bp *vapBC-1* operon. A 1,264 bp kanamycin resistance cassette from pUC4K was ligated into the linearized plasmid, resulting in pDD748. To construct the 86-028NP *vapBC-1* mutant, a high-fidelity PCR product was amplified from pDD748 with the primers BCXbaFor and BCXbaRev and used in MIV transformation. The deletion of the *vapBC-1* locus was confirmed by PCR and DNA sequencing.

For the Δ*vapXD* mutant construction, a three-step cloning strategy was used. First, an upstream (573 bp) region of *vapXD* gene from 86-028NP genomic coordinates 540086–540579 was amplified by high-fidelity PCR with the primer pair 86vapXSacFor (5^′^-ACAGGAGCTCAACTACTCCGTAAA-3^′^) and 86vapXXbaRev (5^′^-CCCGTCTAGATTAATACAGCCTGTT-3^′^). The DNA fragment cut with *SacI* and *XbaI* was cloned into pBluescript II SK(+) cut with *SacI* and *XbaI*, resulting in pDD778. A downstream (619 bp) region of *vapXD* gene from 86-028NP genomic coordinates 541002–541621 was amplified by high-fidelity PCR with the primer pair 86vapDPstFor (5^′^-CGAACTGCAGATTTGCCTAGATAAGCC-3^′^) and 86vapDKpnrev (5^′^-ATAAGGTACCAGCAGCGCTTCACTACC-3^′^). This fragment was cut with *PstI* and *KpnI* was cloned into pDD778 cut with *PstI* and *KpnI*, resulting in pDD786. Then, a 1,348 bp chloramphenicol resistance cassette obtained from pUCΔE*cat* was subsequently cloned into pDD786 cut with *BamHI* to form pDD788. To construct the 86-028NP Δ*vapXD* mutant, a high-fidelity PCR product amplified from pDD788 with the primers 86vapxSacFor and 86vapDKpnRev was used in MIV transformation as previously described [42]. The deletion of *vapXD* was confirmed by PCR and DNA sequencing.

To construct the Δ*vapBC-1* Δ*vapXD* double mutant, the genomic DNA of 86-028NP Δ*vapXD* was used to transform the 86-028NP Δ*vapBC-1* mutant. The 86-028NP Δ*vapBC-1* Δ*vapXD* double mutant clones were selected on chocolate agar plates with both kanamycin and chloramphenicol. The positive clones were characterized by PCR for both deletions using the genomic DNAs of the positive candidates as the template, and verified by DNA sequencing of the amplicons.

### Heterodimerization assays

VapB-1 and VapC-1: for these assays, *vapB-1* was fused to either the LexA DNA binding domain (DBD) in the vector pSR658, resulting in pDD866, or to the LexA DBD of pSR659, resulting in pDD867 [31]. Likewise, *vapC-1* was fused to either the LexA DBD in the vector pSR659, resulting in pDD868, or to the LexA DBD in pSR658, resulting in pDD859. Because the copy number of each plasmid is different, we performed reciprocal assays in which we switched the protein fusions (i.e. from the low copy to the high copy plasmid, and vice versa) as internal controls. Both fused plasmid sets (pDD866 and pDD868, or pDD867 and pDD859) or the unfused vectors (pSR658 and pSR659) were co-transformed and co-expressed in the reporter strain SU202. This strain has a chromosomal construct that consists of a *lacZ* reporter gene controlled by the strong *sulA* promoter, which contains an engineered LexA operator sequence. When there is no fusion to the LexA DBD, the strain constitutively expresses a high level of β-galactosidase. However, if a protein fused to the LexA DBD in pSR658 and another protein fused to the LexA DBD in pSR659 can heterodimerize, a competent LexA dimer is formed that can bind to the engineered LexA operator and repress transcription of *lacZ* in the reporter strain SU202. Homodimers, if formed, cannot bind to the engineered operator site. Expression of the LexA fusion in pSR658 and pSR659 is induced by IPTG, and since β-galactosidase is a very stable enzyme, the reporter strain is routinely grown overnight with IPTG, so that any enzyme that was transcribed prior to induction of the LexA chimera has the opportunity to degrade. This strategy resulted in a more reliable and accurate quantitation of heterodimerization. Following overnight incubation in LB broth with 1 mM IPTG, the reporter strain carrying pSR658 and pSR659, or the LexA DBD fusions, was diluted and grown to log phase in LB broth with 1 mM IPTG. The amount of heterodimerization was quantitated by the repression of *lacZ* activity as indicated by β-galactosidase activity assays and compared to the activity of the reporter strain carrying pSR658 plus pSR659 (no fusion). The algorithm for determining β-galactosidase activity is: [OD_420_-(1.75*OD_550_)/t*v*OD_600_*1000, where t=time of reaction development in minutes, v=volume of sample in milliliters, and OD_600_ is the optical density of the culture at 600 nm [43]. This equation allows normalization of different culture densities for comparison purposes.

VapX and VapD: for these assays, *vapX* was fused to the LexA DBD in pSR658, resulting in pDD882, and to the LexA DBD in pSR659, resulting in pDD883. Likewise, *vapD* was fused to the LexA DBD in pSR659, resulting in pDD884, and the LexA DBD in pSR658, resulting in pDD885. Heterodimerization assays measuring β-galactosidase activity were carried out and quantitated as above. Each pair was analyzed at least three times in triplicate.

### Cloning and purification of VapD, Cat, and VapX

To perform ribonuclease (RNase) activity assays, the *cat* (chloramphenicol acetyltransferase) gene was PCR-amplified from pACYC184 by high-fidelity polymerase and ligated to the *SacI/XhoI*-cut pET24b expression vector, resulting in Cat with a C-terminal polyhistidine tag in pDD689. Similarly, *vapXD* was amplified from 86-028NP genomic DNA and ligated to the *SacI/XhoI*-cut pET24b expression vector, resulting in VapD with a C-terminal polyhistidine tag in pDD791. Finally, *vapX* was amplified from 86-028NP genomic DNA and ligated to the *SacI/XhoI*-cut pET24b expression vector, resulting in VapX with a C-terminal polyhistidine tag in pDD902. To overexpress each protein for purification, pDD689, pDD791, and pDD902 were grown to logarithmic phase in BL21(DE3) and induced for 3 hours with 1 mM IPTG. Protein isolation from induced cells was performed with the MagneHis protein purification system (Promega, Madison, WI USA) according to the manufacturer’s instructions.

### NTHi growth dynamics

To compare growth dynamics, the 86-028NP wild type strain and the Δ*vapBC-1*, Δ*vapXD*, and Δ*vapBC-1* Δ*vapXD* mutants were re-suspended from chocolate agar plates grown for 18 hours at 37°C in 5% CO_2_ into fresh sBHI broth to an OD_600_ of ~0.1, then 200 microliters of each re-suspension was placed in triplicate into a sterile nontreated flat-bottomed 96 well plate (#351172, BD Biosciences, Bedford, MA, USA). Empty wells were filled with 200 microliters of sterile water to decrease evaporation, and the plate was covered with sterile gas permeable sealing film (#9123-6100, USA Scientific, Ocala, FL, USA). The plate was incubated for 11 hours with shaking at 35°C in a Multiskan FC spectrophotometer (ThermoFisher Scientific, Waltham, MA, USA), and the OD_595_ was read hourly. Two biological replicates and three technical replicates were performed and analyzed by one-way analysis of variance (ANOVA) for independent samples.

### Transmission electron microscopy (TEM) of NTHi strains co-cultured with EpiAirway tissues

Primary human respiratory epithelial tissues grown at the ALI, the EpiAirway model (MatTek #AIR-100-ABF, Ashland, MA USA) was used for co-culture with NTHi [32]. Each 0.6 cm^2^ tissue was fed basally by 1 ml of the proprietary antibiotic-free maintenance medium, AIR-100-MM-ABF (MM) and cultured at 37°C with 5% CO_2_. Each insert was washed daily with 200 μl of pre-warmed Dulbecco’s PBS (D-PBS) with calcium and magnesium and the basal MM was renewed daily. NTHi strains were grown overnight on chocolate agar plates at 37°C with 5% CO_2_. Bacteria were then suspended in D-PBS to an OD_600_ of ~0.2, and diluted to the desired inoculum. The tissues were inoculated apically with ~1.0 × 10^7^ colony forming units (CFU) in ~25 microliters per insert with the 86-028NP parent strain or the Δ*vapBC-1*, Δ*vapXD*, and Δ*vapBC-1* Δ*vapXD* mutants. On day 5 after infection, the tissues were fixed with 1.25% glutaraldehyde and 2.0% paraformaldehyde in 100 mM sodium cacodylate buffer (pH 7.2) for 24 hours. The membranes were then removed from the plastic inserts using a #11 pointed scalpel blade, processed by the Georgia Health Sciences University Electron Microscopy Core for TEM and imaged with a JEOL JEM 1230 transmission electron microscope (JEOL, Peabody, MA USA) to identify the morphology of the bacteria.

### Quantitation of NTHi inside infected EpiAirway tissues

The EpiAirway tissues at the ALI (#AIR-100-ABF, MatTek, Ashland, MA USA) were infected apically with the suspensions of either the 86-028NP parent strain, or the Δ*vapBC-1*, Δ*vapXD*, and Δ*vapBC-1* Δ*vapXD* mutants individually at ~10^7^ CFU per insert (n = 6). The inoculation suspensions were quantified by dilution and plating for viable colony counting. The inserts were washed and the basal MM renewed daily. On day 1, 2, 4, 6 and 8 after infection, each insert was harvested as previously described [32]. Briefly, each insert was washed with D-PBS, then 300 μl of MM with gentamicin (100 μg/ml) was added apically to each insert, with 1 ml of MM with gentamicin (100 μg/ml) added basally. After 1 h of incubation at 37°C with 5% CO_2_, the inserts were washed 3X with D-PBS without calcium and magnesium, and 250 μl of 1% saponin in D-PBS without calcium and magnesium was added apically to each insert and incubated at 37°C for 10 min. Subsequently, the tissues were harvested, disaggregated and diluted to 1 ml in D-PBS. The suspensions were then diluted serially and plated onto chocolate agar plates for bacterial CFU counts.

### NTHi survival in the chinchilla otitis media model

Healthy female adult (400–600 g) chinchillas were purchased from a commercial supplier and handled in accordance with the recommendations in the Guide for the Care and Use of Laboratory Animals of the National Institutes of Health. The protocol was approved by the Mercer University Institutional Animal Care and Use Committee (Assurance Number: A3725-01). All surgery was performed under isoflurane anesthesia, and all efforts were made to minimize suffering. Animals were allowed to acclimate to the vivarium for 1 week prior to challenge, and none had any visible signs of middle ear infection as detected by otoscopy. The 86-028NP parent strain and the Δ*vapBC-1*, Δ*vapXD*, and Δ*vapBC-1* Δ*vapXD* mutants were recovered from frozen stocks and cultured for 18 h on chocolate agar at 37°C with 5% CO_2_. The bacteria were harvested, suspended in D-PBS containing 0.1% gelatin (D-PBSG), loaded into tuberculin syringes, and maintained on ice for the challenges. Chinchillas were anesthetized by isoflurane inhalation and each middle ear was injected transbullarly with 100 μl (~ 1000 CFU) of bacteria (n = 4 to 5 animals with 8 to 10 middle ears per challenge strain) or D-PBSG alone (control). Actual challenge doses were confirmed by plating followed by colony counting. On day 4 post-challenge, the animals were euthanized by cardiac exsanguination and their superior bullae were opened. Middle ear fluid was recovered, and each middle ear was washed with 1.0 ml of D-PBSG. An aliquot of each middle ear wash was diluted serially and plated on chocolate agar for CFU counts. Additionally, each mutant strain recovered was plated initially on chocolate agar with and without its cognate antibiotic marker to determine if any genetic changes had occurred during middle ear challenge. No significant differences between the numbers of colonies recovered on plates with or without antibiotics were observed (data not shown).

### Histology of chinchilla bullae

Following sacrifice, the chinchilla ears were dissected, fixed with 10% neutral buffered formalin, and decalcified with 5% formic acid. Each ear was cut at the midline in the sagittal plane, and both halves were processed and paraffin-embedded. Step sections of the distal halves were performed and the resulting slides were stained with hematoxylin-eosin (H&E) for analysis. One of us (A.N.W.), a Board-certified pathologist, scored randomized and blinded sections from the same step-sections of each ear for the relative level of the inflammatory response, with the control (buffer only) ears being scored as 0 (no inflammation), with the most inflammation being designated as 4+.

### Ribonuclease (RNase) activity assays

Varying amounts of purified VapD, VapX, or Cat (chloramphenicol acetyltransferase) proteins were incubated at 37°C for 30 minutes with 25 pmol of RNaseAlert substrate (Integrated DNA Technologies, Coralville, IA) using the manufacturer’s buffer in a final volume of 25 μl. The RNaseAlert substrate is a single stranded RNA with a fluorophore (FAM) on one end and a quencher on the other. When cleaved, the substrate fluoresces brightly. This sensitive assay allows us to monitor RNase activity in real time. Negative controls consisted of the MagneHis protein elution buffer with no protein, and 0.6 μg of the Cat and VapX proteins. The reactions were placed in a Bio-Rad white 48-well PCR plate, covered with optical film and incubated in a MiniOpticon thermocycler at 37°C. Plate reads were taken every 5 minutes for 30 minutes. The average relative fluorescence units (RFU) from two independent assays are reported. All solutions used were nuclease free or treated with diethyl pyrocarbonate.

### Statistical analysis

Data are presented as the mean ± standard deviation (SD). Differences among multi-group treatments were determined by one-way ANOVA using the VassarStats website for statistical computation (http://faculty.vassar.edu/lowry/VassarStats.html). *P* values of ≤0.05 were considered significant, with significant differences further analyzed using a Tukey HSD post hoc test.

## Competing interests

The authors declare that they have no competing interests.

## Authors’ contributions

DR drafted the manuscript, performed the mutagenesis, protein-protein interaction, and EpiAirway assays, and participated in the animal studies. ANW performed the histological analysis and edited the manuscript. DAD conceived and designed the study, performed the animal studies and participated in drafting and editing the manuscript. All authors read and approved the final manuscript.
